# 

**Published:** 1897-10-09

**Authors:** 


					THE HOSPITAL. ABSOLUTELY PURE, therefore BEST. Oct. 9th, 1897. C
CADBURY'S gmK M CADBURY'S. .
COCOA IM| IF* COCOA JjIERAR^
""""" no alkaliesGENERAL'S 0F;
soon Western InfSmaix 1 ? 111 1 ' JNMdLJiZiZ/JOHir/cti hosp.lxal -
No. 576. Vol. XXIII. [SS?p?] Saturday,
Oct. 9th, 1897.
[ ?seeptp02o ] PRICE TWOPENCE.

				

